# Appraisal Biases About Strangers in Posttraumatic Stress Disorder

**DOI:** 10.1007/s10608-018-9962-1

**Published:** 2018-11-30

**Authors:** Juliane Sachschal, Elizabeth Woodward, Anke Ehlers

**Affiliations:** 1 Centre for Anxiety Disorders and Trauma, Department of Experimental Psychology, University of Oxford, Old Rectory, Paradise Square, Oxford, OX1 1TW UK; 2 Oxford Health NHS Foundation Trust, Oxford, UK

**Keywords:** PTSD, Person impressions, Negative appraisals

## Abstract

Cognitive theories of posttraumatic stress disorder (PTSD) suggest that negative appraisals play an important role in the development and maintenance of PTSD. It has not yet been examined experimentally how people with PTSD appraise strangers. Twenty-two trauma survivors with PTSD and 26 non-traumatised controls completed a person impression updating task. There was no group difference in initial kindness ratings of people shown in the photos. The PTSD group changed their ratings more when receiving negative compared to positive information about the person, relative to controls. When incongruent information was subsequently presented, the PTSD group tended to be more influenced by negative information than positive information, relative to congruent information, than controls. The findings suggest selective attention to negative information about others in PTSD.

**Electronic supplementary material:**

The online version of this article (10.1007/s10608-018-9962-1) contains supplementary material, which is available to authorized users.

## Introduction

Experiencing traumatic events can shatter the fundamental assumptions that people have about themselves, others and the world (e.g., Janoff-Bulman and Frieze 1983). Cognitive theories of PTSD suggest that excessively negative appraisals about the trauma and its sequelae play an important role in the development and maintenance of PTSD symptoms (e.g., Dalgleish 2004; Ehlers and Clark 2000; Foa et al. 1999; Resick and Schnicke 1993). Effective trauma-focused treatments for PTSD target dysfunctional appraisals of the trauma and aim to update the personal meaning of the trauma (Ehlers and Clark 2000; Resick and Schnicke 1993). Analyses of session-by-session changes in appraisals and symptoms suggest that changes in negative appraisals play an important role in recovering from PTSD symptomatology (Kleim et al. 2013; Zalta et al. 2014).

The importance of negative trauma-related appraisals of the self and others, such as “I am inadequate” or “No one can be trusted”, in PTSD has been well documented. Studies have shown that trauma survivors with PTSD reported more negative trauma-related appraisals compared to those without PTSD (e.g., Foa et al. 1999). Furthermore, studies found that endorsement of negative appraisals predicted the subsequent PTSD severity in trauma survivors (e.g., Dunmore et al. 2001; Ehring et al. 2008; Evans et al. 2007; Halligan et al. 2003), and emergency workers assessed before trauma exposure (Bryant and Guthrie 2005; Wild et al. 2016). Together, these results suggest that negative trauma appraisals play a role in the development and maintenance of PTSD symptomatology. However, one limitation of these studies is that trauma-related appraisals were only assessed by self-report.

One group of prominent appraisals in people with PTSD concerns evaluations of other people, such as “All people are bad”, even if they have never met them before. To the authors’ knowledge, it has not been experimentally examined how people with PTSD appraise strangers. The current study draws on the literature on impression updating of strangers to investigate this question. Humans have a motivation to evaluate others with limited information, such as “Are they good or bad people?” (e.g., Piazza et al. 2014; Todorov et al. 2008). This allows them to predict future behaviors of others, and gain control about what to expect in the future (e.g., Uhlmann et al. 2015). A person-centred approach of moral judgement suggests that actions are not only evaluated in terms of whether they are positive or negative, or what consequences they have. They also suggest that actions reflect a person’s moral character, suggesting that actions holding most information about a person’s character are more diagnostic than others (e.g., Uhlmann et al. 2015).

Research on person impressions in social cognition has for a long time applied a two-dimensional framework, suggesting that person impressions are mainly formed on two dimensions, namely warmth and competence (Fiske et al. 2007). Recent research suggests that morality is another dimension that plays an important role in forming impressions of others. Studies found that immoral behaviors are more diagnostic than moral behaviors (e.g., Skowronski and Carlston 1987; Uhlmann et al. 2015; Wojciszke et al. 1993), suggesting that negative actions may be more helpful in predicting future behavior of others. The concept of kindness in person impressions has been suggested to map on the dimension of warmth or sociability (e.g., Brambilla and Leach 2014), but other authors suggest that kindness is an important trait of morality as well (Goodwin 2015). People with PTSD are hypervigilant and sensitive towards negative information, and they may be particularly inclined to evaluate a stranger’s character negatively if they learn that they showed unkind acts.

In everyday life, it is important to adapt current appraisals when new information becomes available. For example, after someone is hurt in a trauma by a stranger and thought that no one was helping them, they may initially appraise this as meaning that other people are bad and unkind. It is important for them to realise that most other people do not harm them and to update their initial appraisal to “most other people are not bad”. People with PTSD may have difficulties with updating such appraisals without intervention. Studies on emotional regulation suggest that PTSD was related to less use of reappraisal strategies (e.g., Ehring and Quack 2010), indicating that people with PTSD may have difficulties in adapting initial appraisals with new information. A general computerised reappraisal training has further been found to reduce the development of intrusions after exposure to traumatic film clips in healthy participants (Woud et al. 2012) and to reduce distress induced by intrusions (Woud et al. 2013). These findings give further support for the importance of appraisal updating in PTSD, and suggest that improving reappraisal strategies in PTSD may help to reduce the development of PTSD symptoms.

To date, it has not been examined experimentally how people with PTSD update appraisals about strangers. Studies on healthy participants found that it is generally more difficult to update negative compared to positive first impressions about others (Mende-Siedlecki et al. 2013a, b). A recent fMRI study further suggests that it does not merely depend on the valence of the stimuli how evaluations are updated but that impressions are more likely to be updated when exposed to information that provides most informational value, for example actions that are unexpected or less frequent (Mende-Siedlecki and Todorov 2016). In PTSD treatment, specific interventions are often needed to update negative cognitions about others such as behavioral experiments and updating the memory of the trauma (Ehlers et al. 2005) as people with PTSD tend to focus on negative aspects that confirm their view of other people. It is therefore expected that people with PTSD are worse in updating initial negative impressions about strangers than other people.

## Aims of the Present Study

The present study used an adapted version of a person impression task (Mende-Siedlecki et al. 2013b). In this paradigm, participants repeatedly rate strangers for a personal attribute (e.g., kindness) in response to negative and positive information about the behavior of that person. Of particular interest are changes in ratings after incongruent information (e.g., description of a positive behavior following a description of a negative behavior or vice versa). This study examined how PTSD influences (1) how positively people appraise strangers, (2) the degree to which negative and positive information affects how strangers are appraised, and (3) how appraisals about strangers are changed with new information. It was hypothesised that compared to controls, participants with PTSD would (1) rate strangers as less kind, (2) change their kindness rating of strangers more when receiving negative compared to positive information about them, and (3) show smaller changes in their kindness ratings after receiving incongruent positive information than with incongruent negative information.

## Methods

### Recruitment

The study received ethical approval by the the Medical Sciences Inter-Divisional Research Ethics Committee and the NRES Committee South Central - Oxford C under the reference number 14/SC/0198. Recruitment started in June 2014 and ended in April 2016. Non-traumatised participants were recruited via circular emails to students and staff of the University of Oxford, and through adverts on several participant recruitment websites. Participants with PTSD were recruited from participating National Health outpatient services in London and Oxford. General inclusion criteria were age between 18 and 65 years, being able to read and write in English, no history of psychosis, and no current substance dependence. The PTSD group met DSM-5 diagnostic criteria for PTSD as determined by the Clinician-Administered PTSD Scale for DSM-5 (CAPS; Weathers et al. 2013). The control group was screened to have no previous exposure to a traumatic event according to DSM-5 diagnostic criteria. Participants who responded to the advert and met diagnostic criteria for a current diagnosis of PTSD were included in the PTSD group (n = 4) along with the treatment seeking PTSD patients. People who expressed an interest in the study were sent a full information sheet via email, and given at least 48 h to consider their decision to take part.

### Participants

Twenty six non-traumatised participants and 22 participants with PTSD were invited to attend a 2-h research session. Sample characteristics are displayed in Table 1. Traumatic experiences in participants with PTSD included road traffic accidents (n = 4), sexual assault (n = 4), witnessing other people die/getting seriously hurt (n = 3), childhood trauma (n = 2), sudden traumatic death of significant other (n = 3), non-sexual assault/armed robbery (n = 2), other traumatic events (n = 4). The PTSD and control group did not differ in age, *t*(44) = − 0.76, *p* = .45, years of education, *t*(46) = − 0.40, *p* = .69, gender, *X*^*2*^ = 1.64, *p* = .18, and ethnicity, *X*^*2*^ = 2.64, *p* = .11. As expected, the groups differed in symptom severity of posttraumatic stress disorder, *t*(46) = 5.65, *p* < .001, and depression, *t*(45) = 7.66, *p* < .001.

**Table 1:** Demographics and symptom scores of PTSD and control group

	PTSD (*n* = 22)				Control (*n* = 26)			
	Mean	SD	N	%	Mean	SD	n	%
Age (years)	31.46	9.72			33.35	20.16		
Years of education	16.75	3.23			17.42	2.76		
Ethnicity								
Caucasian			18	72			25	96
Ethnic minority			4	28			1	4
Gender								
Male			9	41			5	19
Female			13	59			21	81
PDS (trauma/negative control event)	35.91	13.01			7.52	8.81		
BDI (n = 46)	21.95	8.93			4.92	5.99		

*PDS* Posttraumatic Diagnostic Scale, *BDI* Beck Depression Inventory-II

### Impression Updating Task

#### Stimulus Material

Four parallel picture sets and eight behavior sets (four parallel positive, four parallel negative) were created. Each picture set contained 15 photos of neutral faces derived from the AR Face Database (Martinez and Benavente 1998). The sets were balanced for gender (7 male, 8 female) and ethnicity (7 white, 8 non-white) of the individuals displayed on the pictures. The pictures were randomly paired with behaviors during the task, and each of the behaviors only occurred once. Behavior sets contained 15 sentences introducing the name of the individual (neutral), 15 sentences describing a 15 negative (non-trauma) and 15 sentences describing a positive behavior ascribed to the person shown in the photos (e.g., ‘[name] spat at someone’ or ‘[name] volunteered in a homeless shelter’). Behaviors were adapted from Fuhrmann et al. (1989). The sets of sentences describing positive and negative behaviors used in the task can be found in Table 4. In the development phase of the material, 15 people completed an online survey to rate behaviors for their kindness. The different sets of positive and negative behaviors were matched for kindness ratings (see Table 2).

**Table 2:** Mean kindness ratings for all behavior sets

Behaviors	Kindness ratings		
	Mean	SD	Range
Negative			
Set 1	2.19	0.86	1.52–2.76
Set 2	2.19	0.86	1.32–2.80
Set 3	2.20	0.89	1.52–2.84
Set 4	2.20	0.86	1.52–2.76
Positive			
Set 1	5.56	0.79	5.16–6.44
Set 2	5.56	0.79	5.24–6.16
Set 3	5.56	0.79	5.12–6.04
Set 4	5.56	0.79	5.12–6.28

#### Stimulus Presentation

The task was programmed on Matlab, Version R2013b. Stimuli were presented on the screen of a 21.5″ iMac. Photos of faces and sentences describing behaviors were presented within a black frame. Faces were presented with a size of 15 × 10 cm and behaviors were presented underneath the picture so that they were easy to read. Each consisted of 15 trials, so participants completed a total of 60 trials. The 60 targets were presented in a randomised order, with the constraint that the same condition could be presented no more than three times in a row.

**Fig. 1:**
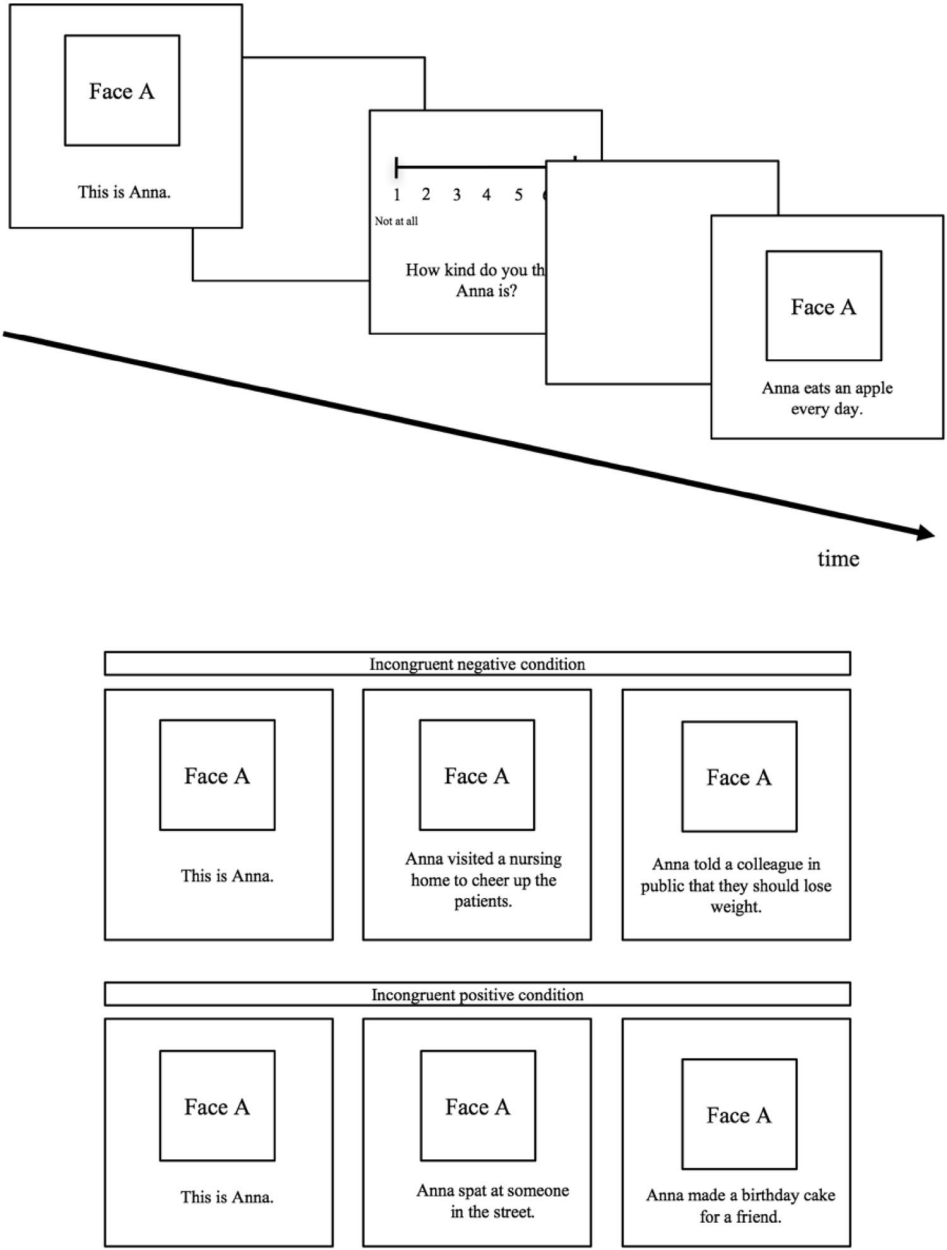
Example items for stimuli sequences in the incongruent positive–negative and negative–positive conditions and example of stimulus presentation. Photos and sentences were presented for 3 s. Afterwards, a white screen appeared, followed by the rating scale. The photo of each person was first shown with neutral information (their name) and then subsequently paired with two different behaviors, and was rated for kindness three times

Participants were first presented with a picture of a person that introduced their name (neutral information, e.g., ‘This is Anna’; t1). Second, participants were presented with the same picture, paired with a positive or negative behavior (t2). Third, participants were presented with the same picture a third time with either a congruent (positive if positive at t2, or negative if negative at t2) or incongruent (positive if negative at t2, or negative if positive at t2) behavior (t3). Participants completed two practice trials before starting the actual task. Figure 1 displays an example of how stimuli were presented. Pictures and paired behaviors appeared for three seconds. The rating scale advanced automatically when participants gave their response.

#### Experimental Design

Picture and behavior sets were paired randomly across the four different conditions: (1) neutral–negative–positive (incongruent negative–positive), (2) neutral–negative–negative (congruent negative), (3) neutral–positive–negative (incongruent positive–negative), (4) neutral–positive–positive (congruent positive). Within each condition, all behaviors were sorted by the mean kindness ratings from the online survey so that for the two incongruent conditions the mildest negative behavior was always paired with the mildest positive behavior, and the strongest negative behavior always paired with the strongest positive behavior. Similarly, for the two congruent conditions, the mildest negative/positive behavior was always paired with the mildest negative/positive behavior, respectively.

#### Dependent Variable

Participants rated how kind the person shown in the photo was three times, i.e., after t1, t2 and t3, on a 7-point Likert scale from 1 = *not at all* to 7 = *very much so*. Change scores for changes in kindness ratings were calculated as the modulus of the difference between t1 and t2, and between t2 and t3.

#### Pilot Study

The task was piloted to test whether the adapted version of the task could replicate the pattern of results from the original task which presented behaviors of the same valence twice for each condition (Mende-Siedlecki et al. 2013b). Results are reported in the supplemental material.

### Symptom Measures

#### **Posttraumatic Diagnostic Scale** (PDS; Foa et al. 1997)

The PDS assesses the severity of PTSD symptoms. Subjects are asked to rate each of the symptoms for a DSM-5 diagnosis on a scale from 0 = *not at all* / *only one time* to 3 = *5 or more times a week* / *almost always*. The sum score of all items is the total severity score. The 17-item version of the PDS for DSM-IV was administered with four additional items added for the DSM-5 (Foa et al. 2015). Cronbach’s alpha in the current sample was α = .96.

#### **Beck Depression Inventory-II** (BDI-II; Beck et al. 1996)

Severity of depression symptoms was assessed with the BDI, a standardized and validated measure of depressive symptoms over the past 2 weeks. Each of the 21 items lists four different statements representing different symptom severity levels, and participants indicate which one applies best to them. The BDI has been found to have high internal consistency (Cronbach’s α = .91) and showed good correlation with other depression inventories (Beck et al. 1996).

### Questionnaires

#### General Information Questionnaire

This questionnaire assessed demographic characteristic (age, sex, ethnicity, education), see Table 1.

### Procedure

Participants were invited for a 2-h research session. Participants gave written informed consent after the nature of the study had been fully explained. Patients with PTSD had completed the CAPS as part of their clinical assessment. Other participants were screened for history of trauma to assure that participants had not experienced an event classified as traumatic by DSM-V criteria (DSM-5 American Psychiatric Association 2013). At the start of the session, participants filled in the BDI and PDS with regards to their traumatic event, or for their most distressing non-traumatic event (control group). Participants then completed some memory-related tasks, including an adapted version of a picture location updating task (Novak and Mather 2009), an adapted version of a questionnaire on memory qualities (Halligan et al. 2003), and the digit span task (Wechsler et al. 2008). The findings will be reported elsewhere. At the end of the session, participants completed the impression updating task. Participants were reimbursed for their time and travel with £20.

### Data Analysis

To test the first hypothesis (participants with PTSD rate strangers as less kind than other participants), kindness ratings for the initial neutral picture presentation at t1 were compared with a univariate ANOVA with the between-subject factor group (PTSD, controls). To test the second hypothesis (participants with PTSD change their kindness ratings more after negative information than after positive information, and this difference is greater than for control participants), a mixed-measures ANOVA compared the change score in kindness ratings from t1 to t2 with the within-subject factor valence (negative, positive), and the between-subject factors group (PTSD, controls). To test the third hypothesis (participants with PTSD show smaller changes in their kindness ratings after receiving incongruent positive information than with incongruent negative information, and this difference is greater than for controls), a mixed-measure ANOVA compared change scores in kindness ratings from t2 to t3 with the within-subject factors valence (negative, positive) and congruency (congruent, incongruent), and the between-subject factors group (PTSD, control).

## Results

### Initial Ratings of Strangers

The univariate ANOVA did not show a main effect of group, *F*(1, 46) = .60, *p* = .44, η_p_^2^ = .01, indicating that there were no differences in how the groups rated the people shown in the photos at t1, contrary to Hypothesis 1. Table 3 displays raw scores for kindness ratings at all time points.

**Table 3:** Mean raw scores for kindness ratings in PTSD and control groups for all time points

	PTSD		Controls	
	Mean	SD	Mean	SD
Rating t1	4.21	0.71	4.09	0.26
Rating t2				
Negative	2.61	0.50	2.80	0.43
Positive	5.26	0.70	5.31	0.46
Rating t3				
Incongruent				
Neg–Pos	4.23	0.90	4.28	0.61
Pos–Neg	3.04	0.64	3.44	0.70
Congruent				
Neg–Neg	1.98	0.45	2.14	0.59
Pos–Pos	5.83	0.77	5.85	0.58

Neg–Pos = incongruent from negative behavior at t2 to positive at t3. Pos–Neg = incongruent from positive behavior at t2 to negative at t3. Neg–Neg = congruent negative at t2 and t3. Pos–Pos = congruent positive at t2 and t3

### Influence of Negative and Positive Information on Kindness Ratings

**Fig. 2:**
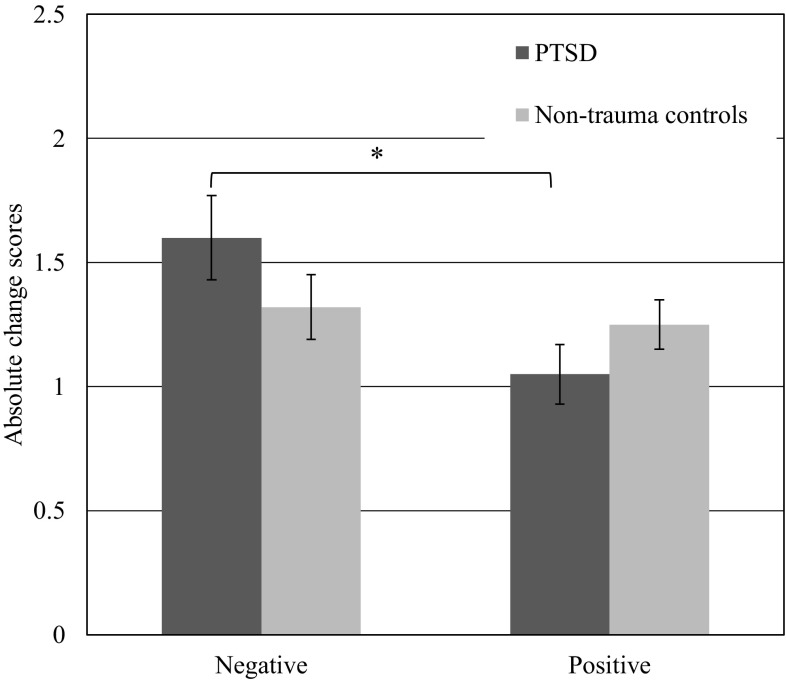
Mean change scores (modulus of difference scores) in kindness ratings about strangers from t1 (neutral information) to t2 (negative or positive information) by group. Change scores for negative information represent a decrease in kindness ratings, while change scores for positive information represent an increase in kindness ratings

Mean change scores from t1 to t2 for the PTSD and control groups by valence are displayed in Fig. 2. The mixed-measure ANOVA showed a significant main effect of valence, *F*(1, 46) = 7.51, *p* < .01, η_p_^2^ = .14, indicating that kindness ratings generally changed more after the presentation of negative information compared to positive information. There was no significant main effect of group, *F*(1, 46) = 0.18, *p* = .66, η_p_^2^ = .004, but there was a significant valence x group interaction, *F*(1, 46) = 4.28, *p* = .04, η_p_^2^ = .09, indicating that the PTSD and control groups showed different patterns in the way they adjusted their kindness ratings after receiving negative or positive information. Participants with PTSD had greater change scores for negative than positive behaviors, *t*(21) = 2.49, *p* = .02, *d* = 1.09, whereas the control group did not show a valence effect, *t*(25) = 0.98, *p* = .33, *d* = 0.29. Separate group comparisons for each valence were not significant. As shown in Fig. 2, the direction of the group differences for negative and positive behaviors was in the opposite direction. For negative behaviors, the PTSD group showed (non-significantly) greater change scores than controls, *t*(46) = 1.56, *p* = .13, *d* = 0.45, and for positive behaviors, controls showed (non-significantly) greater change scores than the PTSD group, *t*(46) = 1.23, *p* = .23, *d* = 0.35. Hence, in line with Hypothesis 2, the PTSD group adjusted their ratings more when receiving negative compared to positive information about strangers, relative to the control group.

### Influence of Congruent and Incongruent Information on Kindness Ratings

**Fig. 3:**
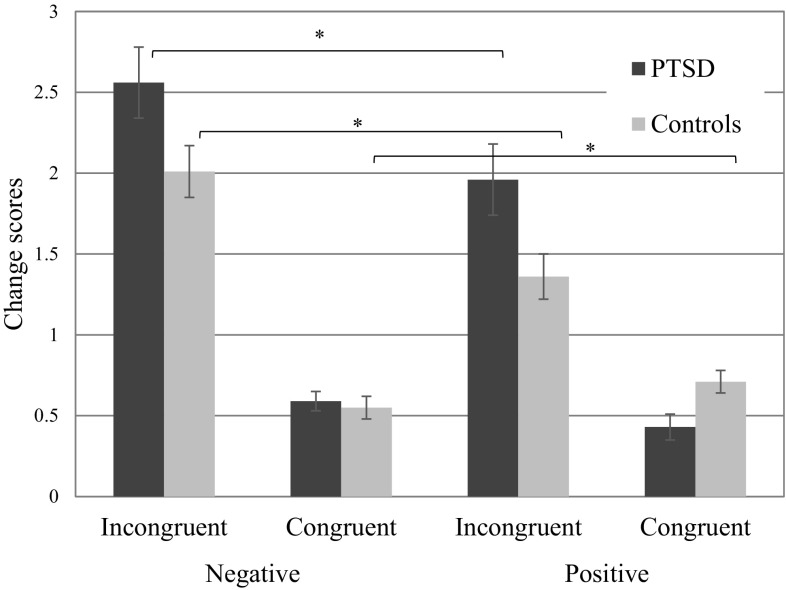
Changes in kindness ratings from t2 to t3 after incongruent and congruent negative and positive information. In the incongruent condition, participants received either positive information at t2 and negative information at t3 (negative incongruent condition), or negative information at t2 and positive information at t3 (positive incongruent condition). In the congruent condition, participants either received negative information at t2 and t3 (negative congruent condition), or positive information at t2 and t3 (congruent positive condition)

Mean change scores after congruent and incongruent negative or positive information (t2–t3) are displayed in Fig. 3. There were main effects of congruency, *F*(1, 46) = 67.82, *p* < .001, η_p_^2^ = .60 and valence, *F*(1, 46) = 24.98, *p* < .001, η_p_^2^ = .35, and a valence × congruency interaction, *F*(1, 46) = 24.38, *p* < .001, η_p_^2^ = .35, indicating that participants showed greater changes in ratings after negative than after positive information, and that this difference was larger in the incongruent condition. There was also a trend for a 3-way interaction between group × valence × congruency, *F*(1, 46) = 3.45, *p* = .07, η_p_^2^ = .07. Separate ANOVAs by group found significant valence × congruency interactions for participants with PTSD, *F*(1, 21) = 24.75, *p* < .001, η_p_^2^ = .54, and for controls, *F*(1, 25) = 4.63, *p* = .04, η_p_^2^ = .16, indicating that both groups showed greater change scores for negative compared to positive incongruent information, relative to the congruent condition, but that this effect was greater in the PTSD group. The group × valence effect was tested for each condition separately. In the incongruent condition, change scores were greater for negative behaviors than positive behaviors in both the PTSD group, *F*(1, 21) = 16.60, *p* < .001, η_p_^2^ = .44, and the control group, *F*(1, 25) = 14.21, *p* = .001, η_p_^2^ = .36. For the congruent condition, there was no valence effect in the PTSD group, *F*(1, 21) = 0.50, *p* = . 49, η_p_^2^ = .02, and a greater change for consistent negative compared to positive behavior in the control group, *F*(1, 25) = 6.38, *p* = .02, η_p_^2^ = .20. When each of the four conditions were considered separately, no group differences in change scores were observed, *p* values between .18 and .77. To sum up, in line with Hypothesis 3, both groups more readily updated their ratings when receiving incongruent negative compared to incongruent positive information. In contrast to Hypothesis 3, this effect was not more pronounced in the PTSD compared to the control group.

## Discussion

The study examined experimentally how trauma survivors with PTSD and non-traumatised control participants appraise strangers, how negative and positive information about these strangers influences these appraisals, and how appraisals are adjusted after receiving incongruent information about them. People with PTSD did not generally appraise strangers more negatively than the control group. However, relative to controls, they appraised strangers more negatively after receiving negative compared to positive information about them. Finally, relative to controls, the PTSD group also tended to have more difficulties in updating initial negative appraisals when they received positive information following negative information. However, this effect did not reach significance. The findings indicate that there were no general appraisal biases in PTSD towards strangers but that negative information about strangers had more impact on evaluations about strangers in participants with PTSD and may be more difficult to update. More research is needed to further explore these findings.

Contrary to Hypothesis 1, the groups did not differ in their initial appraisals of strangers they saw in photos, indicating that people with PTSD did not generally perceive strangers more negatively than the control group. This is surprising as people with PTSD have been found to have excessive negative appraisals about the world and others in self-report measures (e.g., Foa et al. 1999). Due to the little amount of research on this topic in the PTSD literature, it can only be speculated about potential reasons for this finding. Firstly, it is conceivable that people with PTSD may not perceive strangers as less kind, but may perceive them as more dangerous or less trustworthy. Research suggests that morality plays an important role in person evaluations (e.g., Brambilla et al. 2011), and it has been suggested that social judgements are influenced on different dimensions, such as sociability and morality. In the posttraumatic cognition inventory (Foa et al. 1999), the scale on negative cognitions about the world mainly comprises items on trust and danger, such as ‘People cannot be trusted’, referring to the moral aspects of person impressions. Kindness has been suggested to form part of the warmth dimension (e.g., Brambilla and Leach 2014), which may explain the discrepant findings between this and other studies. Secondly, people with PTSD may appraise strangers to be likely to behave negatively when it comes to interactions related to their own person. For example, people with PTSD tend to blame themselves for what happened to them, and tend to have a negative view about themselves (Foa et al. 1999). A better understanding of different aspects in cognitive appraisals about the self and others in PTSD may be needed to understand appraisal biases in PTSD more generally. Finally, participants were asked to give the rating immediately after receiving the information about the stranger. Self-report measures, on the other hand, assess appraisals about others retrospectively. One of the strongest cognitive predictors for the development of PTSD after a traumatic event is rumination (e.g., Dunmore et al. 1999; Ehlers et al. 1998; Ehring et al. 2008). Appraisal biases about strangers in PTSD may be developed through rumination after the actual event already happened. It might be interesting to measure appraisals after a time delay in future studies to better understand the influence of time on appraisals in PTSD. Overall, more research is needed to explain the lack of a general appraisal bias of strangers in PTSD in this study.

In line with Hypothesis 2, relative to controls, trauma survivors with PTSD changed their initial appraisals of strangers more readily after receiving negative compared to positive information. These findings indicate that even though people with PTSD did not show a general negative appraisal bias towards strangers (Hypothesis 1), they showed an appraisal bias in response to negative compared to positive information about strangers relative to controls. This suggests that people with PTSD may more readily than other people develop negative appraisals about strangers when these are associated with negative information. In treatment, it may therefore be helpful to focus on positive behaviors of others to help divert the attention from negative towards more positive attributes in others. The findings add to recent studies on attention bias modification that suggest that training PTSD patients to disengage from threatening material may help reduce PTSD symptomatology (e.g., Beard et al. 2012; Hakamata et al. 2010).

The study did not show that within the control group, negative information about strangers influenced the kindness ratings more than positive information at t2. This finding is different from the effects observed in the pilot study (see supplemental material) and the previous literature which has shown that healthy participants respond more strongly to negative compared to positive information (e.g., see Wojciszke 2005 for review). The discrepant findings may be explained with the adaptation of the task in this study. In previous studies, the influence of negative or positive information was assessed after two presentations of consistent negative or positive behaviors. In this study, this effect was replicated in the congruent condition at t3 where the control group showed a greater change in kindness ratings after having received two times negative compared to two times positive information about a stranger. This suggests that the effect of negative information on ratings may be better replicable with two consecutive presentations. Future studies should use the extended paradigm with two consecutive trials of consistent information in order to establish the negativity bias.

In line with Hypothesis 3, relative to controls, people with PTSD tended to be better in updating initial appraisals with negative compared to positive information. Both, PTSD and control group had more difficulties in updating appraisals with positive compared to negative information. The findings replicate previous studies on person impression-updating showing that healthy participants have more difficulties updating initial negative impressions with positive information than vice versa (e.g., Mende-Siedlecki et al. 2013b). However, a recent study suggests that updating may not be dependent on the valence of the incongruent information that people receive but rather depend on predictability (Mende-Siedlecki and Todorov 2016). This interpretation would suggest that the control group expected positive behavior and therefore updated better when they received negative information. The PTSD group showed the same pattern, which could be seen as counter-intuitive, as people with PTSD may expect negative behavior and be more surprised to find out that someone behaves in a positive way. There are some possible explanations for this. Mende-Siedlecki and Todorov (2016) argue that in healthy participants, unexpected or unpredictable events with lower base rates are seen as more valuable and are therefore more likely to update previous information. It is conceivable that people with PTSD experience negative information as more valuable for survival due to their past experiences even though they are to be expected. It is also possible that the initial negative appraisal of strangers is stronger as it is diagnostically valuable and therefore is more difficult to be updated. More research is needed to better understand these findings.

The study has several limitations. Firstly, the study used non-traumatised participants as a control group. This means that it is not possible to determine whether the effects found in this study can be attributed to PTSD or trauma in general. Furthermore, the study also did not use a control group of participants with depression, and did not assess comorbid disorders. It is therefore unclear whether the effect found in this study may be attributed to high depression scores in the PTSD group. Secondly, the appraisals assessed in this task were dissociated from the self in that participants only rated photos of people, and not people that they interacted with. It is possible that participants tried to give objective, rational kindness ratings, rather than rate their emotional and initial response. Other variables, such as trustworthiness, may be more sensitive to biases in PTSD and more likely to detect potential differences and should be included in future studies. Thirdly, it is possible that the negative behavior sets used in this study were perceived as more intensively negative than the positive behavior sets were perceived as positive. The mean ratings of the negative behaviour sets used in this study were further away from the numerical midpoint than the positive behavior sets, *t*(118) = 3.91, *p* < .001. We did not test the perceived intensity directly and do not have ratings for neutral behaviours that would allow us to determine the perceived midpoint of the scale. Nevertheless, we cannot rule out that participants responded more strongly to negative compared to positive information due to the strength of the behaviour sets. Thus, the main effects of valence observed in this study should be interpreted cautiously. However, the effects of main interest to this study were differences between the PTSD and control groups and between congruent and incongruent information, which remain unaffected. Fourthly, participants did not rate the photos in this task without the sentence stating their name. As the sentences with names and behaviors were randomised to the photos, this would however have minimised error variance. Fifthly, the behaviors used in this task could be interpreted as being relevant for other concepts than kindness, for example trustworthiness. It is therefore difficult to say whether the ratings were only related to kindness. Finally, the impression updating task assessed appraisals right after the information became available. The process of forming and updating appraisals right after receiving information may differ from the process of maintaining these reappraisals over time. It would therefore be interesting to assess changes in appraisals after completing filler tasks, or once new information has entered working memory.

In conclusion, people with PTSD did not appraise strangers differently from controls, but showed a negative appraisal bias when they learned negative information about them. PTSD was not associated with greater difficulties in updating information in general, but relative to controls, the PTSD group tended to show greater differences between updating incongruent negative and positive information, compared to congruent information. More research is needed to better understand impression-updating in PTSD. Results may help better understand how updating appraisals contributes to the maintenance of PTSD symptoms in the aftermath of trauma.

### Electronic supplementary material

Below is the link to the electronic supplementary material.

Supplementary material 1 (DOCX 69 KB)

## Appendix

See Table 4.

**Table 4:** Negative and positive behavior sets adapted from Fuhrman et al. (1989)

Set 1—negative
Scratched a stranger’s car with her/his key
Pulled the seat out from underneath somebody, so they fell
Kicked a puppy that came too close to her/him
Refused to help a child find their parents
Ridiculed a person behind their back
Shoved at a man who was passing out leaflets
Insulted someone speaking about human rights
Let the dog go to the toilet on her/his neighbour’s garden
Broke a someone’s camera and never replaced it
Dropped rubbish out the window of her/his moving car
Pushed in front of someone in a bus queue
Cut out and stole articles from library journals
Refused to pay the service charge in a restaurant
Refused to take a phone message for a colleague
Did not want to lend her/his car to a friend who needed it
Set 2—negative
Spat at someone in the street
Deliberately turned in someone else’s project under her own name
Smoked in a no-smoking area even though others complained
Refused to hold the door for someone in a wheelchair
Tricked someone to subscribe to a non-existent magazine
Did not show up for a date and never called to cancel
Ignored a new person in the office for several weeks
Threw some rubbish in someone else’s garden
Refused to make a cup of tea for a visiting friend
Closed the elevator door before anyone else could get on
Continuously interrupted others in a conversation
Took her/his neighbour’s newspaper
Walked by someone who had dropped their shopping and did not help
Refused to help her/his parents to clean out the storage room
Refused to attend the funeral of her/his disliked relative
Set 3—negative
Intentionally drove through a puddle, splashing some pedestrians
Told a colleague in public that they should lose weight
Kicked a stray cat to get it to leave her/his yard
Laughed at a person who tripped and fell and injured themselves
Shouted an insult at a stranger in the street
Pushed someone when trying to get onto an escalator
Did not give her/his seat to a pregnant woman on the bus
Refused to talk to a friend who needed support
Shouted at someone at the self-checkout for being too slow
Gave out several cheques to an empty account
Returned a birthday gift because it was too cheap
Borrowed someone’s favourite book and never gave it back
Started an argument with a co-worker about nothing
Didn’t reply to an email from a friend asking for advice
Refused to take over a colleague’s shift even though it was an emergency
Set 4—negative
Started a false rumour about someone
Swore at a cashier who made an error
Spat off the balcony at the theatre and laughed about it
Kicked a dog for eating cheese from the kitchen floor
Turned someone in to the police for not paying their TV licence
Stole an expensive camera from a shop
Insulted the parents of a good friend
Refused to help clean the dishes after a friend’s dinner party
Yelled at her/his neighbours for having an afternoon garden party
Shouted at someone for disagreeing in a conversation
Refused to lend some tools to her/his neighbour
Didn’t share her/his birthday cake with any colleagues
Slammed a door in someone’s face at work
Didn’t call back her/his friend who had called five times
Refused to help a friend by proof reading an important letter
Set 1—positive
Accompanied refugees to the authorities to help fill in their paperwork
Visited a nursing home to cheer up the patients
Offered to help an elderly neighbour to paint their walls
Did someone’s food shopping to help them out
Accompanied a worried friend to the doctor
Found an expensive briefcase and tried to locate its owner
Participated in an effort to clean up a city park
Collected a prescription for an elderly neighbour
Helped an old lady to cross the road
Offered to help someone carry a suitcase up the stairs at the train station
Gave her/his balloon to a child who had let theirs go
Held the bus door open for a person running to get the bus
Sponsored a work colleague in a fundraising event
Sent a package to a friend who had recently moved away
Brought gifts from a holiday for their friends
Set 2—positive
Stood up for a colleague that other people were bullying
Refused to gossip about a colleague
Volunteered time at an orphanage, taking children on day trips
Helped push someone’s car out of a snow bank
Brought chocolate to her/his friend who was ill
Made dinner for her/his friend who recently broke up with their partner
Babysat the baby of a friend
Paid for a friend’s dinner
Went out of her/his way to offer someone a lift home
Made a birthday cake for a friend
Picked up the dry cleaning for a neighbour
Gave her/his seat to someone on the crowded bus
Lent someone her/his phone to make a phone call
Sent a thank you card to a colleague that had helped them out
Helped someone to carry their luggage to the car
Set 3—positive
Shared his/her lunch with a homeless person
Volunteered several hours a week at a runaway shelter
Gave someone with heavy bags a lift home from the supermarket
Helped a lost child find their parents in a store
Helped a man in a wheelchair cross a busy intersection
Helped someone with their tax return
Included someone in a conversation who was sitting alone
Helped a neighbour clear out their garage
Posted a letter that was found in the street
Visited a sick friend in the hospital
Cleaned someone else's dirty dishes in the sink
Fed her neighbours' cat while they were on holiday
Won a stuffed animal at a fair and gave it to a child
Walked the dog for an acquaintance
Sent a personal card to all friends at Christmas
Set 4—positive
Helped to set up a night shelter in the community
Spent time voluntarily tutoring disadvantaged students
Collected winter clothes to give to charity
Lent money to a friend in a financial crisis
Brought an elderly person home
Helped a friend move to a new flat
Mowed the lawn for their elderly neighbour
Donated books to a nursing home
Volunteered to stay late to help a co-worker
Put the bins out when her/his neighbour was away
Listened to a friend who had difficulties at work
Shared an umbrella with someone during the rain
Bought someone flowers
Sent a birthday card to a friend that had not been in touch for a while
Picked up a locked bike that had fallen on the side walk
