# Supplementary material for: Appraisal Biases About Strangers in Posttraumatic Stress Disorder
Source: Cognit Ther Res. 2018 Nov 30;43(1):247–58. doi: 10.1007/s10608-018-9962-1 (PMC6514061; doi:10.1007/s10608-018-9962-1)
Supplement: Supplementary file 1 — Supplementary material 1 (DOCX 69 KB) [file 10608_2018_9962_MOESM1_ESM.docx]

**Supplemental material**

The task was piloted with 24 (16 female, 8 male) healthy participants to test whether the pattern of results from the original task could be replicated. Participants were excluded if they reported a mental health problem or the experience of an interpersonal trauma. Paired t-tests compared the change scores from t1 to t2 after negative and positive information. A trend for a significant valence effect was found, *t*(23) = 2.04, *p* = .05, indicating that participants showed greater absolute change after receiving negative compared to positive information about a stranger. Paired t-tests then compared the change from t2 to t3 after incongruent and congruent information. Participants showed significantly less change after incongruent positive compared to negative information, *t*(23) = 3.33, *p* = .003, indicating that it is more difficult to update negative impressions with positive information than vice versa. There was no valence effect for change scores after congruent information, *t*(23) = 1.35, *p* = .19, indicating that appraisals were adapted similarly for negative and positive congruent information.
